# Lipid-Based Nanocarriers in Renal RNA Therapy

**DOI:** 10.3390/biomedicines10020283

**Published:** 2022-01-26

**Authors:** Chi-Ting Su, Daniel H. W. See, Jenq-Wen Huang

**Affiliations:** 1 Department of Medicine, National Taiwan University Cancer Centre, Taipei 10672, Taiwan; chitingsu@gmail.com (C.-T.S.); neodeoxys1@gmail.com (D.H.W.S.); 2 Department of Human Genetics, Graduate School of Public Health, University of Pittsburgh, Pittsburgh, PA 15213, USA; 3 Renal Division, Department of Internal Medicine, National Taiwan University Hospital Yunlin Branch, Douliu 640, Taiwan

**Keywords:** renal disease, RNA therapy, RNAi, miRNA, siRNA, nanoparticle, lipid nanocarrier

## Abstract

Kidney disease is a multifactorial problem, with a growing prevalence and an increasing global burden. With the latest worldwide data suggesting that chronic kidney disease (CKD) is the 12th leading cause of death, it is no surprise that CKD remains a public health problem that requires urgent attention. Multiple factors contribute to kidney disease, each with its own pathophysiology and pathogenesis. Furthermore, microRNAs (miRNAs) have been linked to several types of kidney diseases. As dysregulation of miRNAs is often seen in some diseases, there is potential in the exploitation of this for therapeutic applications. In addition, uptake of interference RNA has been shown to be rapid in kidneys making them a good candidate for RNA therapy. The latest advancements in RNA therapy and lipid-based nanocarriers have enhanced the effectiveness and efficiency of RNA-related drugs, thereby making RNA therapy a viable treatment option for renal disease. This is especially useful for renal diseases, for which a suitable treatment is not yet available. Moreover, the high adaptability of RNA therapy combined with the low risk of lipid-based nanocarriers make for an attractive treatment choice. Currently, there are only a small number of RNA-based drugs related to renal parenchymal disease, most of which are in different stages of clinical trials. We propose the use of miRNAs or short interfering RNAs coupled with a lipid-based nanocarrier as a delivery vehicle for managing renal disease.

## 1. Introduction

The global prevalence of chronic kidney disease (CKD) is estimated at approximately 13.4%, while estimates suggest that acute kidney injury (AKI) affects approximately 13.3 million people globally every year [1,2]. According to the Global Burden of Disease study in 2017, 1.2 million deaths were attributed to CKD, and it was the 12th leading cause of death worldwide [3]. CKD is defined as the presence of renal damage (usually detected as a urinary albumin excretion of >30 mg/day) or decreased renal function (characterised by an estimated glomerular filtration rate (eGFR) of <60 mL/min/1.73 m^2^) for over three months, irrespective of the cause [4]. The persistence of renal damage or decreased function for at least three months is necessary to distinguish CKD from AKI. Conversely, the occurrence of AKI is abrupt and the decline in GFR is usually reversible [5]. Taking into consideration the aforementioned concerns, the employment of RNA therapy is being looked at for the management of renal diseases as its use in other diseases such as cancer and in vaccinations has gained traction. Some of the major advantages of RNA therapy over other therapies include its capacity to target an otherwise non-targetable small molecule and its ability to rapidly interact with messenger RNA (mRNA) constructs [6].

Over the years, studies on the diverse functions and mechanisms of action of RNA molecules have enabled its use in therapy. From the discovery of mRNA in 1961 [7] to the approval and use of RNA-based drugs, the research on RNA has indeed come far. Along the same timeline, but in a different field, is the progression of nanotechnology. Following the initial introduction of the concept of nanotechnology by American physicist and Nobel Prize laureate Richard Feynman in 1959, the use of nanotechnology is now seen in almost every field of science, including the field of medicine [8]. One of the current uses of nanotechnology in medicine is its application in drug delivery.

Both the RNA-based drug development and efficient drug delivery methods have advanced substantially and simultaneously to allow for the clinical use of RNA therapy. Most recently and notably in this regard, are two authorised vaccines used against the coronavirus disease 2019 (COVID-19) [9]. Both the BNT162b2 and mRNA-1273 vaccines employ lipid nanocarriers to facilitate the delivery of antigenic mRNA [10,11]. In this review, we aim to examine the role of RNA in renal pathophysiology and its putative role in the pathogenesis of renal parenchymal diseases. We also discuss various strategies for using short interfering RNA (siRNA) and microRNA (miRNA) as therapies and the process of drug delivery. The advantages of using lipid-based nanocarriers as efficient delivery systems of RNA molecules are elaborated. The review will focus mainly on the clinical applications of RNA therapy for renal diseases.

## 2. RNA Therapy

It is widely accepted that RNAs are important molecules involved in nearly all biological pathways [12]. While the diverse roles of RNA were being discovered, it was also noticed that the use of RNA as a form of therapy had substantial potential. The current principle of RNA therapy involves the use of both coding and non-coding RNAs. There are five known types of therapeutic RNAs that are classified by their mode of action: (1) RNAs that inhibit RNA activity (miRNAs, siRNAs, antisense RNAs), (2) RNAs that target proteins (RNA aptamers), (3) RNAs that reprogram genetic information (trans-splicing ribozyme), (4) RNAs that encode therapeutic proteins (mRNAs) and (5) DNA-modifying CRISPR guide RNAs (gRNAs) [13]. The initial use of RNA as a form of therapy employed chemically synthesised antisense oligonucleotides [12]. In addition, the discovery and research of interference RNA (RNAi) has led to vast possibilities in the field of RNA therapeutics. RNAi uses siRNAs or miRNAs. Both antisense RNA and RNAi work by suppressing the expression of a target RNA [12].

Of particular interest here are the RNAs that relate to protein downregulation, which are miRNA and siRNA. As miRNAs and siRNAs have emerged as a class of therapeutics with great potential, especially for the treatment of cancer, there has been some interest in using them as a therapy for renal diseases [14,15]. Both miRNAs and siRNAs are powerful tools that can silence post-transcriptional gene expression. For example, a specifically designed miRNA/siRNA targets an mRNA via sequence-specific binding, resulting in degradation of mRNA translation [14]. Current application in cancer therapy uses synthetic siRNA to target oncogenes and genes involved in cancer cell proliferation, survival, invasion, angiogenesis, metastasis and drug resistance [14]. A look into how these applications of siRNAs translate to similar renal therapies would prove highly useful. Studies in animal models have shown that siRNA uptake is rapid and effective in kidneys without affecting basic renal function parameters [16]. Furthermore, miRNAs have been discovered to be implicated in many diseases, including renal disease, as these components are often dysregulated in the disease process [17]. Thus, there is potential in exploiting the involvement of miRNAs in the pathogenesis of disease towards discovering new treatment options. Moreover, as miRNAs are highly effective tools for gene silencing, the possibility of developing gene-specific RNA drugs is vast, including for genes with no currently known pharmacological antagonists or inhibitors [15]. It is, therefore, not surprising that the application of miRNAs and siRNAs would be worth tapping into and should be extensively researched and applied to renal therapy.

## 3. Rationale of RNA-Based Strategy in Renal Diseases

### 3.1. Renal Fibrosis

In various progressive renal diseases, TGFβ signalling plays an essential role. In human studies, TGFβ1 expression is majorly upregulated in fibrotic tissues [18]. Accumulating evidence has demonstrated that miRNAs regulate the TGFβ pathways [19,20]. Conversely, TGFβ also affects miRNA expression [21,22,23]. Micro-150 promoted renal fibrosis in a renal ischemia/reperfusion model [24]. Similarly, increased miR-668 [25] and miR-489 [26] expression was observed in acute kidney injury (AKI) in humans, mice, and renal tubular cells by hypoxia-inducible factor-1 (HIF-1) signalling. In addition, abundant extracellular matrix protein deposition is a key feature of fibrosis. The expression of collagen types I and III was affected by mmu-miR-29-5p [27]. TGFβ reduces miR-29-5p expression, leading to the feedforward upregulation of TGFβ expression [28]. In addition, it has been documented that miR-21 promotes epithelial-to-mesenchymal transition (EMT) induced by TGFβ1 by inhibiting the target Smad7 [29].

### 3.2. Diabetic Nephropathy

Podocyte apoptosis has been documented to be associated with diabetic nephropathy, and miRNAs have been implicated in the pathogenesis of diabetic nephropathy. The expression of mmu-miR-29c increased in glomeruli and microvascular endothelial cells in a mouse model [30]. Studies of renal tissues of patients with diabetes revealed that miR-192 expression was inversely related to renal fibrosis and was associated with eGFR [31,32]. Other studies have revealed that TGFβ is involved in miR-192 regulation in proximal convoluted tubule cells exposed to high-glucose conditions [31]. TGFβ upregulated the expression of miR-192 and consequently inhibited the expression of Zeb1/2 which sequentially increased the expression of miR200 [33]. Hyperglycaemia induced cell hypertrophy and increased matrix protein levels in mouse diabetic models, and miR-21 manipulated this process by reducing tumour suppressor protein phosphatase and tensin homologue deleted on chromosome 10 (PTEN) expression [34]. Overall, the impact of miRNAs on these functions is important.

Downstream effects of TGF-β are mediated by connective tissue growth factor (CTGF), whose expression is upregulated in several renal cells and secreted in the urine in the diabetic state [35]. In addition, the interaction of advanced glycation end products (AGEs) with their receptors (RAGE) generates oxidative stress and stimulates fibrotic reactions in diabetic kidneys [36,37]. Furthermore, PTEN is a tumour suppressor protein that is recognised as a negative factor of the phosphoinositide-3-kinase (PI3K) Akt signalling pathway [38]. Evidence has revealed that miR-214 regulates PTEN expression via Akt activation, leading to high glucose-induced mesangial and proximal tubular cell hypertrophy and fibronectin deposition [39].

### 3.3. Glomerulonephritis

Glomerulonephritis includes a group of diverse disorders that may present as proteinuria or microscopic haematuria with or without renal dysfunctions. Accumulating evidence has revealed that RNA-related pathophysiology and potential molecules or pathways may be therapeutically useful for treating glomerulonephritis.

IgA nephropathy is caused by abnormal O-galactosylation of IgA, resulting in the deposition of IgA complexes in the kidneys and activation of the complement system. Various miRNAs play roles in IgA nephropathy as observed in a genome-wide analysis [40]. Members of the miR-200 and miR-29 families, which regulate EMT and progressive renal fibrosis, caused prominent expression changes in IgA nephropathy and were associated with renal fibrosis and proteinuria. Patients with IgA nephropathy presented increased expression of miR-146a and miR-155 compared to controls. These two factors were inversely associated with eGFR, and proteinuria and the latter were related to renal fibrosis [41]. miR-155 has been reported to affect T cell development and reduce regulatory T cell number and T cell dysfunction, which is associated with the pathogenesis of IgA [42,43,44]. Another study reported that the inhibition of miR-21 expression potentially reduced fibrogenic processes in podocytes and tubular cells by preventing PTEN/Akt pathway activation [45].

Systemic lupus erythematosus (SLE) is a systemic disease caused by abnormal immune system activities. Kidney biopsy analysis of patients with lupus nephritis revealed upregulated miR-146 and miR-198 expression in glomerular lesions and increased miR-638 expression in tubulointerstitial lesions [46]. Interstitial miR-638 expression was associated with clinical markers of proteinuria, while miR-146 expression was associated with eGFR. Thus, these two factors are pathogenic factors for the development of clinical lupus nephritis. In addition, miR26a and miR-30b were reported to be involved in cell cycle regulation and their reduced expression was observed in renal tissues as well as in a human mesangial cell model [47]. In lupus nephritis, increased expression of human epithelial growth factor receptor (HER-2) and the degree of HER-2 amplification was associated with lupus activity and the severity of proteinuria. It was revealed that INFꭤ enhanced HER-2 expression, which led to miR-26a and miR-30b upregulation and proliferative lupus nephritis.

Focal segmental glomerulosclerosis (FSGS) is characterised by the obliteration of the capillary lumen and increase in mesangial matrix amount in the involved glomeruli. Podocyte injury is a factor affecting the development of FSGS, and molecules associated with increased permeability of glomerular basement membranes are potentially related to the pathogenesis of FSGS [48,49]. MicroRNA-30 has been postulated to maintain cytoskeletal integrity and podocyte health [50]. The growth of podocyte cells is reported to be regulated by the expression of miR-132 and miR-134 [51]. In addition, serum and urine miRNAs have been used as biomarkers to evaluate FSGS disease activity. Lower concentrations of miR-125 and miR-186 have been detected in patients with disease remissions [52]. FSGS disease activity is reported to be associated with miR-196 and miR-30 expression [53]. The latter was a potential predictor of steroid responsiveness in patients with active FSGS.

### 3.4. Hypertension

Hypertension is a significant risk factor for coronary artery disease, congestive heart failure, sudden death, left ventricular hypertrophy and stroke [54,55,56]. Hypertension is the second most common cause of end-stage kidney disease in the US and in a study, the prevalence of hypertension in CKD group was higher than that in control group [57]. It is a complex disease with different factors involved in its pathogenesis, including genetic, environmental, haemodynamic, and renal factors. MiRNAs are involved in pathophysiological alterations related to the progression of hypertension and cardiovascular and renal complications.

Activation of the renin-angiotensin-aldosterone system (RAS) plays an essential role in the pathophysiology of hypertension. The expression of many miRNAs, including miR-29b, miR-129-3p, miR-132, and miR-212, are upregulated by Ang II in HEK293N cells [58]. In addition, Ang II type 1 receptor (AT1R)-regulated miR-483 serves as a potential negative regulator of RAS components in vascular smooth muscle cells (VSMCs) [59].

Oxidative stress potentially induces endothelial cell apoptosis and atherosclerosis development. Overexpression of miR-210 inhibits apoptosis and reduces ROS levels [60]. Inhibition of miR-1555 increases endothelial nitric oxide synthase (eNOS) expression and NO production, restoring endothelium-dependent vasorelaxation in human internal mammary arteries [61].

In addition, sympathetic nervous system overactivity has a significant role in hypertension development and persistence. In genetically hypertensive mice (BPH/2J), the roles of the sympathetic nervous system (SNS) and RAS are important. Hypertension in BPH/2J is mediated by SNS during the active period and RAS during the inactive period [62]. During the active periods, the hypertensive mice had higher Ren1 (renin) mRNA and lower miR-181 expression. Similarly, in a human study, miRNA-181 expression inversely regulated Ren1 mRNA expression in the renal tissue of patients with hypertension [63].

### 3.5. Autosomal Dominant Polycystic Kidney Disease (ADPKD)

ADPKD is a genetic disorder caused by mutations in either PKD or PKD2 genes, resulting in impaired ciliary functions, leading to renal and hepatic cyst formations. In a rat model of ADPKD, the expression of miR-21 was found to be upregulated [64]. Inhibition of miR-21 expression reduces cyst growth in a mouse model of ADPKD [65]. In addition, the expression of genes belonging to the miR-17 cluster is upregulated in mouse models and deletion of miR-17 leads to the resolution of cysts and better renal and animal survival [66].

### 3.6. Alport Syndrome

Mutation in genes coding ꭤ3, ꭤ4 or ꭤ5 chains of collagen Type IV leads to abnormal basement membrane formations in the kidneys, eyes, and inner ears. miR-21 is typically expressed in the tubulointerstitial compartment instead of the glomerular compartments in normal mice, while it is expressed equally in both compartments in Col4 ꭤ3-/-mice [67].

## 4. Nanocarriers for Systemic Delivery

The stability of RNA, delivery efficiency, and activation of the innate immune system are the various challenges for the widespread use of RNA therapy. The limitations of RNA-based therapies are attributed mainly to their short half-life and rapid renal clearance [14]. However, recent advancements in nanotechnology have opened more avenues for improving RNA therapies. Nanocarriers have been shown to overcome many challenges associated with the use of systemic RNAs.

Nanotechnology is an interdisciplinary field that can be defined in various ways. Yet, a reasonable general consensus exists, where nanotechnology is concerned with materials and processes taking place in the nanoscale [68]. According to the National Nanotechnology Initiative (NNI) in the United States, nanotechnology is defined as a science, engineering or technology conducted at the nanoscale level which is typically cited between 1 to 100 nanometre (nm) [8]. Nanotechnology has broad applications in medicine, such as drug delivery, gene therapy, drug synthesis, imaging, and diagnostics of diseases [69]. In the field of nanotechnology, one of the materials produced is known as a nanoparticle. Nanoparticles are a wide class of particulate substance ranging between 1 and 100 nm in size [70]. Depending on their size and surface properties, nanoparticles exhibit different functionalities [71]. Nanoparticles are divided into various broad categories based on their morphology, size, and chemical properties. Some of the well-known classes of nanoparticles include carbon-based nanoparticles, metal nanoparticles, ceramic nanoparticles, semiconductor nanoparticles, polymeric nanoparticles and lipid-based nanoparticles [72].

Nanocarriers use agents like nanoparticles as a transport carrier. A carrier system is defined as technology that optimises the therapeutic efficiency of a bioactive compound [73]. This is particularly useful especially in the drug delivery system. Of the various nanocarriers available out there, the one that is of greatest interest is the use of lipid-based nanocarriers as a means of drug delivery. Lipid-based nanocarriers or nanoparticles (LNPs) have various favourable properties, making them an ideal choice. LNPs are characteristically spherical and possess a lipid solid core with a matrix containing soluble lipophilic molecules. As cell membranes are mainly composed of lipids and phospholipids, lipid-based nanocarriers as a drug transport have the natural advantage of better interaction with cells, and therefore facilitating better cellular uptake of transported drug [74]. Furthermore, lipid nanoparticle materials are biocompatible and biodegradable [75]. Several notable classes of lipid-based nanocarriers have been studied for RNA delivery, including liposomes, solid lipid-based nanoparticles, nanostructured lipid carriers, lipidoid nanoparticles, lipophilic conjugates, transfersomes, lipospheres and nanoemulsions. [14,76,77]. It is important to note that there are many other lipid-based carrier systems out there; however, Table 1 summarises some of them and their advantages.

LNPs no doubt aid in the efficient delivery of RNA drugs to their target. Nevertheless, there are still considerations and precautions to be taken. Lipotoxicity has been one of the concerns with using LNPs. Newer generations of LNPs have managed to overcome this issue [82]. Another major consideration is the targeting selectivity of LNPs. Unmodified LNPs have limitations in terms of being able to efficiently reach their intended target to deliver their payload [83]. As LNPs naturally accumulate in the liver, effective targeting strategies have to be employed to ensure successful uptake in other cell types [84]. Addition of specific ligands to the surface of LNPs have so far aided in their selective targeting followed by uptake by intended cells [85]. In the process of formulation design, production, and characterisation of LNPs, some parameters to consider include type of LNP, size, thermal properties, encapsulation efficiency, loading capacity and long-term storage stability [86]. With regards to kidneys, certain properties of LNPs have a huge bearing on targeting, such as size and charge [87]. For example, smaller LNPs penetrated the kidneys more readily; however, they were associated with higher clearance and lower retention [88]. As the glomerular filtration barrier is charge selective, charges of LNPs can influence their interaction with kidneys. Modifications to LNPs have to be carefully formulated as some additions could have both advantages and disadvantages. For example, PEGylation was used to stabilise LNPs and reduce rapid clearance, yet the presence of PEG on the surface of LNPs activated the immune system via induction of anti-PEG IgM production [89]. In short, for a successful delivery of a drug to occur, the LNP has to protect the RNA from degradation, traffic the drug to its intended target and then facilitate the uptake of said drug by target cells to achieve its desired effect.

## 5. Clinical Therapeutic Application of RNA in Renal Diseases

In the previous sections, we discussed the role of RNA and its types in renal diseases, as well as potential RNA dysregulation. MiRNA-based therapies either inhibit deleterious miRNA or replace a deficient one with a beneficial miRNA. Antagomirs or antimiRs and miRNA antagonists, are single-stranded molecules that can bind to mature miRNAs and block their action. Nanoparticle-based viral vectors express the desired miRNA or siRNAs, which are delivered to the target site [90]. In addition, designing an effective RNA-related therapeutic agent is required to avoid degradation and unintended effects, as well as to deliver its payload efficiently to the intended target tissues.

Some phase I and phase II trials are ongoing or have been completed to evaluate the efficacy of RNA-based therapeutic agents for the management and treatment of chronic hepatitis C, diabetes mellitus type 2 with fatty liver and cancers [90,91,92]. Table 2 summarises key studies investigating RNA therapy in renal diseases. In addition, some are used as therapeutic agents for renal diseases. Some studies have revealed the feasible use of siRNA-based therapy to reduce glomerular sclerosis in a mouse model of glomerulonephritis by manipulating TGFβ signalling, resulting in Mapk1 silencing [93]. Furthermore, siRNA against Smad4 prevented renal fibrosis in an in vivo study [94]. Other studies have revealed the efficacy of siRNA-based therapies targeting p53 and meprin-1β, which are essential for apoptosis and depolarisation, in cisplatin-related AKI models [95]. In unilateral ureteral obstruction models, siRNA therapy against Cd40 significantly reduced inflammation and enhanced repair [96]. siRNA-based therapies in the kidney serve as fantastic platforms with promising results, especially for the prevention of AKI, and various clinical trials are ongoing [15].

In contrast to siRNAs, miRNAs typically bind with partial complementarity and usually promote translational repression by triggering mRNA decay, and different miRNAs can interact with the same transcript by overlapping or non-overlapping sites [103]. Importantly, miRNAs play a significant role in the negative regulation of post-transcriptional gene expression. We have previously discussed various miRNAs that have been reported in AKI, renal fibrosis, ADPKD and progression to CKD [38,50,51,52,53,104]. In animal models, evidence has revealed that miRNA dysregulation is a therapeutic target for renal diseases. Chau et al. [105] reported that the inhibition of miR-21 with complementary oligonucleotides reduced renal fibrosis after injury. In a diabetic nephropathy model, an anti-miR-192-based translational approach reduced renal fibrosis and improved proteinuria and is a potential therapy [32]. Several studies have shown that the clinical utility of miRNA mimetics and inhibitors is a promising future intervention for the pathogenesis of renal disease. However, translation of preclinical findings is complicated, and enhancing efficient targeting to the intended tissues is difficult. In addition, various miRNAs are regulated in a cell-type- or organ-specific manner, so off-target and undesired influences in unrelated tissues are high. Some clinical trials are ongoing to investigate miRNA modulators in renal diseases.

Currently, only a few RNA therapies are associated with renal diseases. At the time of writing this article, they are at different stages of clinical trials and have not been approved for clinical use. These include QPI-1002 and NOX-E36 (Emamticap pegol). QPI-1002 uses an siRNA to target the p53 gene and is currently in phase I and II trials for acute renal failure and AKI, respectively [13]. NOX-E36 is an RNA aptamer targeting the C-C motif ligand 2, currently in phase II trials for type 2 diabetes mellitus and albuminuria [6,13]. Another drug is one developed for Alport nephropathy, a genetic disorder characterised by chronic glomerulonephritis that progresses to end-stage renal disease in young adults. An antagomir against miR21 was effectively evaluated in a mouse model, where the animals displayed substantially milder disease and significantly improved survival after treatment [67]. The drug RG012 targeting miR21 is currently undergoing a phase II clinical trial. Another drug is RGLS4326, an antagomir-inhibiting miR17, developed for the treatment of ADPKD, in which hyperproliferation of the renal tubular cells and cyst formation is observed [66]. Treatment with RGLS4326 attenuated cyst growth in several PKD mouse models and human ADPKD models in vitro and is now in a phase I clinical trial. Although also related to other cancers, APN401 is a drug trialled for renal cancer [106]. Patients were treated with siRNA-transfected peripheral blood mononuclear cells to aid in killing cancer cells. The treatment was targeted at patients with metastatic tumours or tumours that could not be surgically removed. This drug has completed phase I clinical trials [102].

## 6. Conclusions and Future Prospects

**Figure 1:**
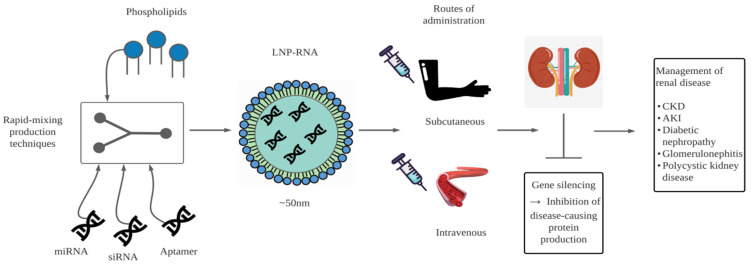
Diagrammatic representation of the packaging and delivery of RNA for the management of renal disease. LNP-RNA, lipid nanocarrier-RNA; CKD, chronic kidney disease; AKI, acute kidney injury.

It is evident in our time that advancements in medicine and technology go hand in hand. As previously mentioned, the world of medicine and therapeutics have benefitted from both advancements in RNA research and the development of nanocarriers. With the constant evolution and mutation of diseases, treatment methods must likewise keep up with disease progression. Therefore, new approaches for managing current diseases are vital. In terms of nephrology, RNA therapy appears to be a novel approach, opening various avenues for the treatment of renal disease (Figure 1).

In this review, we discussed the difficulty in delivering RNA molecules to the intended target. Naked RNAs are unstable in the blood and are rapidly degraded by circulating monocytes and macrophages in the kidneys and liver. Thus, effective delivery methods to prevent miRNA degradation must be developed. The designed delivery vehicles must be non-toxic, have low immunogenicity, and deliver efficiently to the intended targets [107,108]. With this in mind, we propose using LNPs to achieve this goal. With the right formulation in the process of designing LNPs, it is possible to overcome the challenges of using RNA as a viable treatment. As with all treatments, careful attention has to be paid with regards to potential adverse reactions that may occur in RNA therapy. For example, each cellular miRNA is able to regulate the expression of several hundred genes; hence, minor changes anywhere along the miRNA pathway may result in major consequences [109]. In short, off-target effects should ideally be avoided. With the right modifications and appropriate delivery systems, this can be overcome.

Our recommendation is that more emphasis and attention be put on the research of combining RNA with LNPs when it comes to renal disease. There are many types of renal disease out there that could benefit from this research. The use of lipid nanocarrier-based RNA delivery holds a promising future for the treatment of renal disease. We expect to see broad applications of this therapeutics across the board in renal disease in the coming years.

## Figures and Tables

**Table 1:** Summary of the types of lipid-based nanocarriers.

System	Definition	Advantages
Liposomes [14] Cationic lipid-based liposomes Neutral lipid-based liposomes	Liposomes are bilayer structures consisting of hydrated phospholipids. Cationic lipids form complexes or lipoplexes with negatively charged RNA to form nanoparticles by electrostatic interaction. Neutral nanoliposomes are incorporated with RNA to facilitate delivery into cells.	High transfection efficiency Low degradation and high concentration of payload High affinity targeting
Solid lipid-based nanoparticles [14]	Positively charged carriers that include stable nucleic acid-lipid particles (SNALPs) and solid-lipid nanoparticles (SLNs). SNALPs are made from a lipid bilayer containing cationic and fusogenic lipids, stabilised with a diffusible polyethylene glycol-lipid (PEG-lipid) while SLNs consist of natural components of protein-free low-density lipoproteins (LDLs).	Protection against harsh environmental situations Ease of large-scale production
Nanostructured lipid carriers [75,76]	Second generation SLNs formed from a mixture of solid and liquid lipids resulting in an unstructured-matrix due to the different moieties of the constituents.	High drug loading Extended and more controlled drug release
Lipidoid nanoparticles [14]	Complex micelle-like structure of lipid molecules that uses cholesterol and PEG-coating to encapsulate oligonucleotides.	Lower doses of drug required for effect Low toxicity
Lipophilic conjugates [78]	Conjugation of lipophilic molecules such as cholesterol to siRNA.	Higher half-life Higher bioavailability of drug
Transfersomes [79]	Vesicular carriers composed of phospholipids and edge activators.	Efficient transdermal delivery of drugs Able to squeeze into very narrow constrictions (up to 10 times smaller than its diameter)
Lipospheres [80]	Solid lipid core surrounded by phospholipid monolayer.	Administration by non-invasive routes (oral, topical) Effective for poorly water-soluble drugs
Nanoemulsions [81]	Submicron emulsions of two immiscible liquids (oil and water)	Non-toxic and non-irritant in nature Improves bioavailability of drug and reduces dosing frequency

**Table 2:** Summary of clinical trials of RNA therapy in kidney disease.

Name	Type/Target	Disease	Status	Clinical Trial Identifier
QPI-1002 (I5NP)	Short interfering RNA (siRNA) targeting p53 gene	Acute kidney injury Delayed kidney graft function	Phase II trials Phase III trials	NCT02610283 [97] NCT02610296 [98]
NOX-E36 (Emamticap pegol)	RNA aptamer targeting C-C motif-ligand 2	Type 2 Diabetes Mellitus and albuminuria	Phase II trials	NCT01547897 [99]
RG012 (Lademirsen)	Anti microRNA (miR)-21	Alport nephropathy	Phase II trials	NCT02855268 [100]
RGLS4326	Anti miR-17	Autosomal dominant polycystic kidney disease	Phase I trials	NCT04536688 [101]
APN401	siRNA-transfected peripheral blood mononuclear cells	Renal cancer	Phase I trials	NCT02166255 [102]

## Data Availability

Not applicable.
